# Misdiagnosed pneumothorax interpreted as necrotizing fasciitis of the chest wall: case report of a potentially preventable death

**DOI:** 10.1186/1754-9493-8-20

**Published:** 2014-05-05

**Authors:** Lucas S McDonald, Paul G Shupe, Kian Raiszadeh, Anshuman Singh

**Affiliations:** 1 Department of Orthopaedics, Naval Medical Center, 34800 Bob Wilson Drive, Suite 112, San Diego, CA 92134-1112, USA; 2 Department of Orthopaedics, Naval Hospital Jacksonville, 2080 Child Street, Jacksonville, FL 32214, USA; 3 Department of Orthopaedics, Kaiser Permanente Hospital, 4647 Zion Avenue 92120 San Diego, CA, USA

**Keywords:** Pneumothorax, Subcutaneous emphysema, Necrotizing fasciititis

## Abstract

**Background:**

Subcutaneous emphysema is an uncommon clinical finding associated both with benign sources and potentially deadly necrotizing infections. Wide ranges of causes exist including trauma, iatrogenic injuries, factitious disorders and necrotizing infections.

**Case presentation:**

A 49-year old male presented to the emergency room with extensive subcutaneous emphysema following blunt trauma. The orthopaedic surgery service was consulted for treatment of suspected necrotizing fasciitis due to his subcutaneous emphysema. A careful patient history and physical examination correlated with laboratory and radiographic findings revealed rib fractures and a long-standing, undiagnosed pneumothorax as the cause for emphysema. Treatment of the underlying condition with chest tubes led to eventual resolution of the emphysema, though multisystem organ failure ultimately resulted in patient death.

**Conclusion:**

This case illustrates the importance of rapidly and appropriately evaluating trauma patients, and in this case specifically diagnosing and treating the underlying cause of subcutaneous emphysema. The late diagnosis of pneumothorax resulted in delayed definitive treatment, which may have contributed to the patient’s ultimate demise. In acute and sub-acute trauma situations a high level of suspicion for life threatening injuries must be maintained. Decision making for initial treatment should be based on the basic tenants of Advanced Trauma Life Support to primarily address these injuries and help prevent further disability or death.

## Background

Subcutaneous emphysema is a clinical entity with many potential causes ranging from trauma, perforated viscous, breaks in the protective skin barrier and iatrogenic injuries [1-5]. Treatment varies based on the clinical presentation focusing not on the actual emphysema but rather on the underlying cause. One more potentially severe cause of subcutaneous emphysema is a necrotizing infection. Because of the consequences of a delayed surgical treatment for necrotizing infections, many physicians quickly and incorrectly equate subcutaneous emphysema with necrotizing fasciitis. Clinical features of the emphysema combined with individual patient factors help delineate the potential cause and allow for rapid, appropriate treatment. One defining factor of necrotizing fasciitis is its lack of respect for tissue plains with the presence of intramuscular air [6,7]. Misdiagnosis of causal factors leads to a delay in appropriate treatment, whether that is a delay in supportive management, placement of chest tubes, identification of factious disorders or the patient undergoing of unnecessary surgical interventions.

It is crucial to understand presenting factors of necrotizing fasciitis in order to rapidly diagnose and treat the condition or to rule it out and identify the true cause of subcutaneous emphysema. Necrotizing fasciitis is a rare bacterial infection that rapidly destroys skin, muscle, and underlying soft tissue. It typically involves gas-forming bacteria such as Clostridium or Group A *beta*-hemolytic Streptococcus. Because of the expeditious manner at which this infection spreads along deep fascial plains, delay in diagnosis and initiation of treatment can result in devastating outcomes including loss of limb and loss of life. Early, aggressive intervention is necessary to reverse the negative effects of this process and necrotizing fasciitis is a true surgical emergency.

The diagnosis of necrotizing fasciitis is based on clinical suspicion supportive laboratory tests including the Laboratory Risk Indicator for Necrotizing Fasciitis (LRINEC) score as little evidence in the literature supports the accuracy of diagnostic tools [8]. Early presentation may seem innocuous but this is followed by a rapid clinical decline as infection rapidly progresses. Common examination findings include unexpected amounts of pain, progressive skin erythema, soft tissue crepitus, malaise and fever. These findings, however, are nonspecific and symptoms can mimic other conditions. Not all cases of subcutaneous emphysema are due to necrotizing fasciitis and other causes must be kept in the differential diagnosis. While radiographic findings may be impressive, they must be combined with the entire clinical picture including history and physical exam to arrive at the correct diagnosis.

We present the case of a patient with delayed presentation of poly-trauma and associated extremity subcutaneous emphysema prompting urgent orthopaedic consultation for suspicion of necrotizing fasciitis. In this case the extensive subcutaneous emphysema was attributed to rib fractures and a late diagnosis of pneumothorax rather than a necrotizing soft tissue infection.

## Case presentation

A 49-year-old man presented to a community hospital emergency department with a chief complaint of diffuse pain. Over the course of several days, the patient sustained multiple injuries secondary to repeated assaults. He was kicked and punched about the face and chest, a finishing nail was driven under his right great toenail, and his left small and ring fingers had been amputated 8 days prior to presentation. He presented with global pain including his face, thorax, upper and lower extremities. Pain was exacerbated by respiration and associated with shortness of breath.

Prior to this poly-trauma his health was excellent with no significant medical or surgical history. Physical examination in the emergency department revealed a patient in moderate distress but with normal mentation; he was alert and orientated to person, place, and situation. His temperature was 97.6° Fahrenheit, blood pressure was 117/70, pulse tachycardic at 119 beats per minute, and oxygen saturation of 94% on 4 liters of oxygen via nasal cannula. He had significant ecchymosis and abrasions over the right side of the face. Cardiac, pulmonary and abdominal exams were limited due to crepitus and subcutaneous emphysema.

Significant subcutaneous emphysema was present throughout the upper body to include the chest, trunk, extremities, and scrotum. His right upper extremity demonstrated crepitus throughout with swelling, edema and mild tenderness over the wrist and hand but without obvious trauma or penetration of the dermis. There were no overt signs of infection, namely no erythema, warmth, or fluctuance. The left small and ring fingers had been amputated days earlier through the proximal phalanx with non-infectious appearing dry gangrene present distal to a tourniquet. His right great toe had a finishing nail hammered under the toenail and imbedded in the soft tissue without signs of infection.

Laboratory values on admission included an elevated white blood cell count (WBC) of 18.4, C-reactive protein (CRP) elevated to greater than 150 mg/L, creatine elevated to 4.9 mg/dL (433 μmol/L), a blood glucose of 119 mg/dL (6.6 mmol/L), a serum sodium of 125 and a hemoglobin of 11.9. Plain radiographs and computerized tomography (CT) scans demonstrated a mandible fracture, multiple rib fractures with a pneumothorax, left small and ring finger amputations through the proximal phalanx, right great toe with a foreign body, and extensive subcutaneous emphysema throughout the bilateral upper extremities, chest, abdomen and scrotum. No air tracked along the deep fascial planes and there was no intramuscular air.

**Figure 1:**
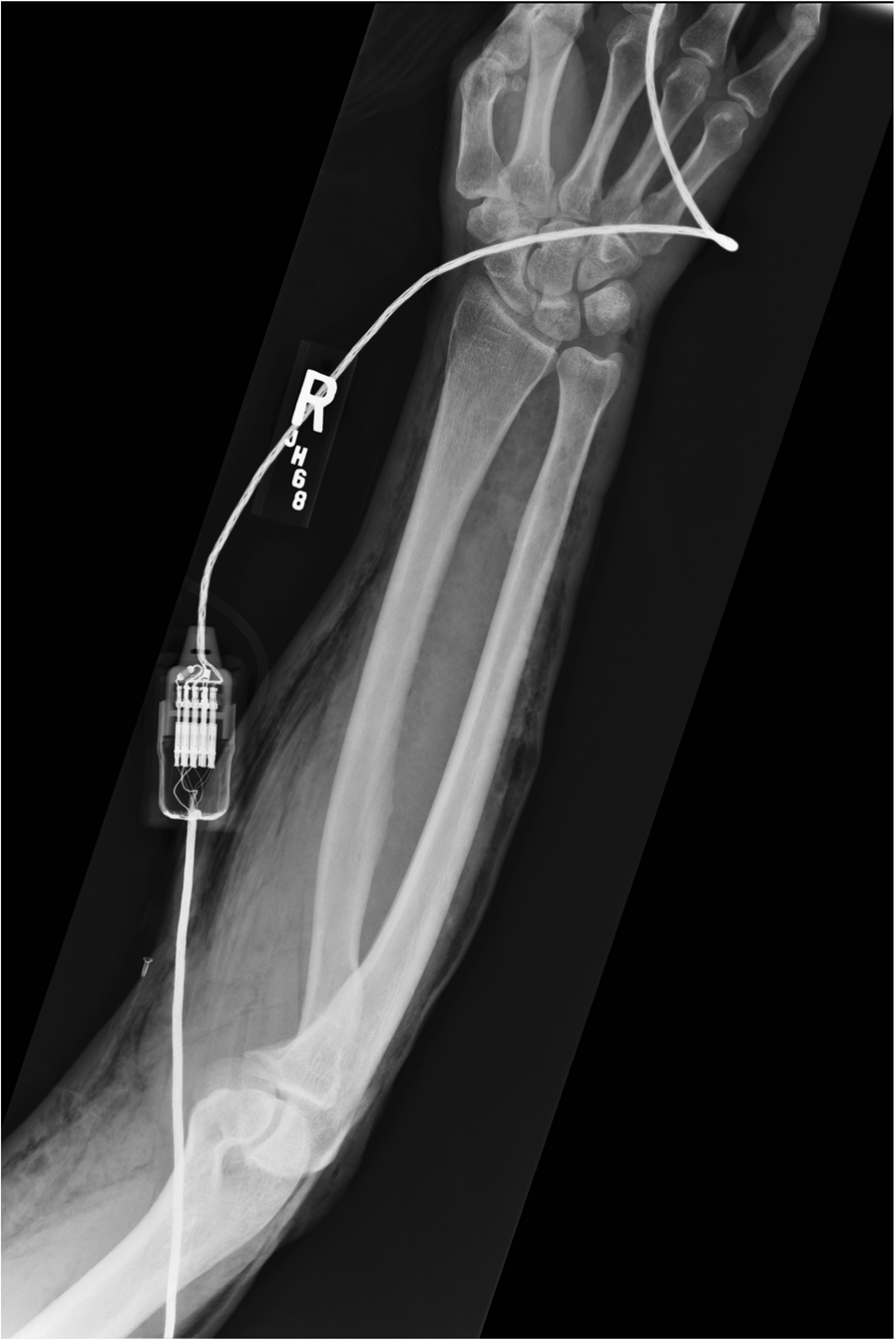
Anterior Posterior view of the right arm taken in the emergency department demonstrating significant subcutaneous free air.

**Figure 2:**
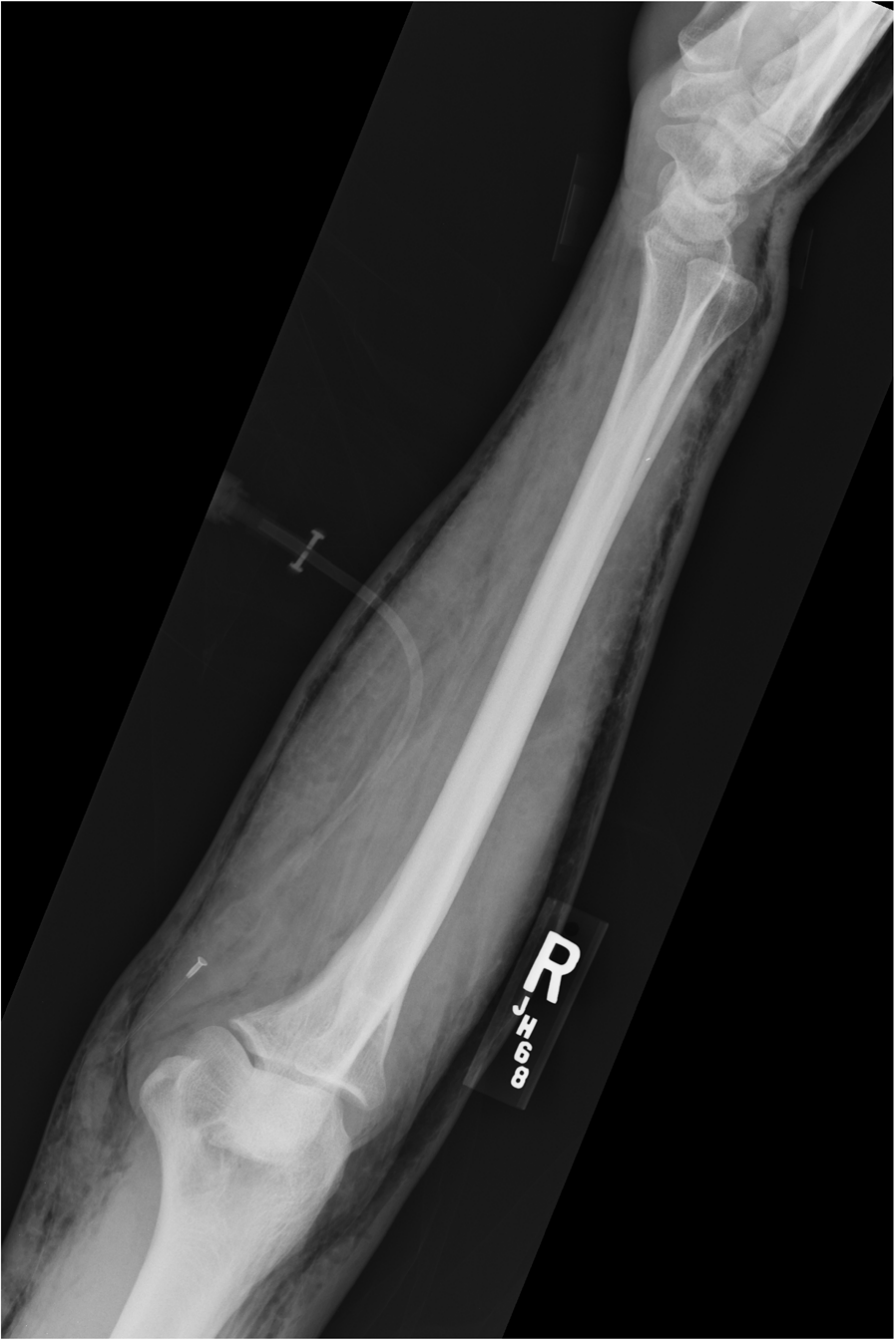
Lateral view of the right arm taken in the emergency department demonstrating significant subcutaneous free air.

**Figure 3:**
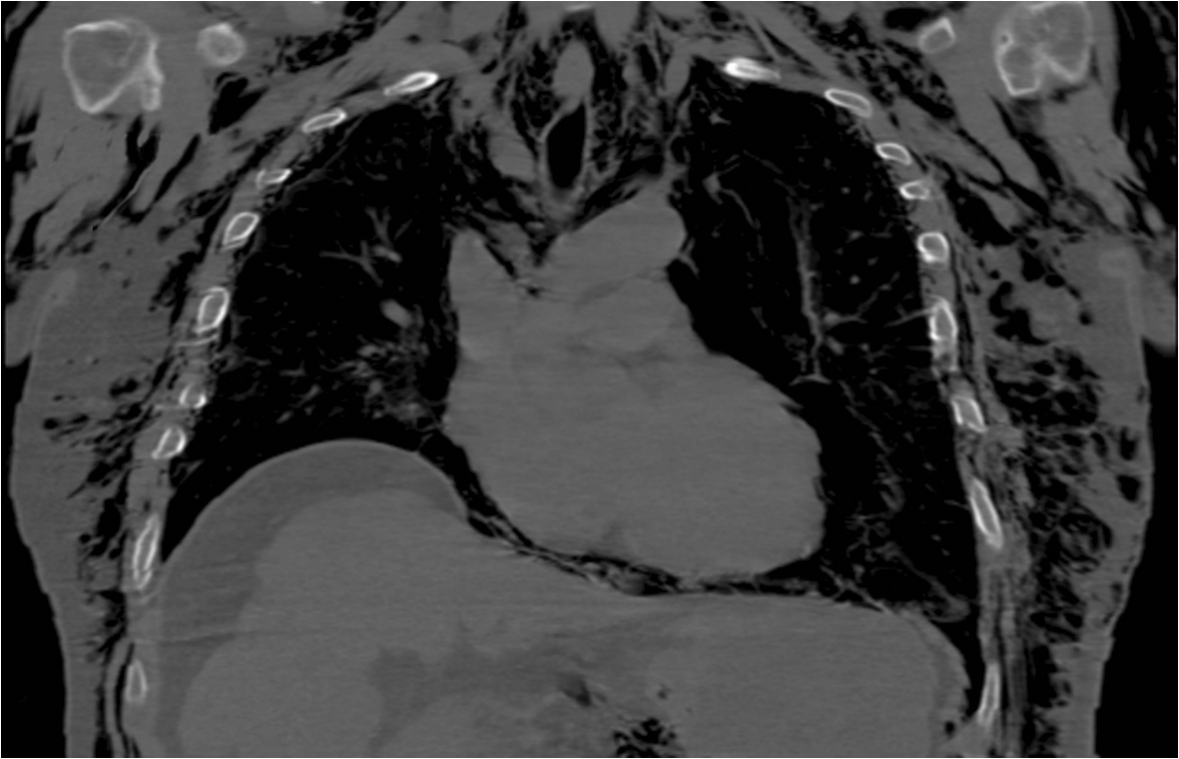
Coronal chest computerized tomography scan demonstrating subcutaneous free air throughout the chest secondary to a pneumothorax.

**Figure 4:**
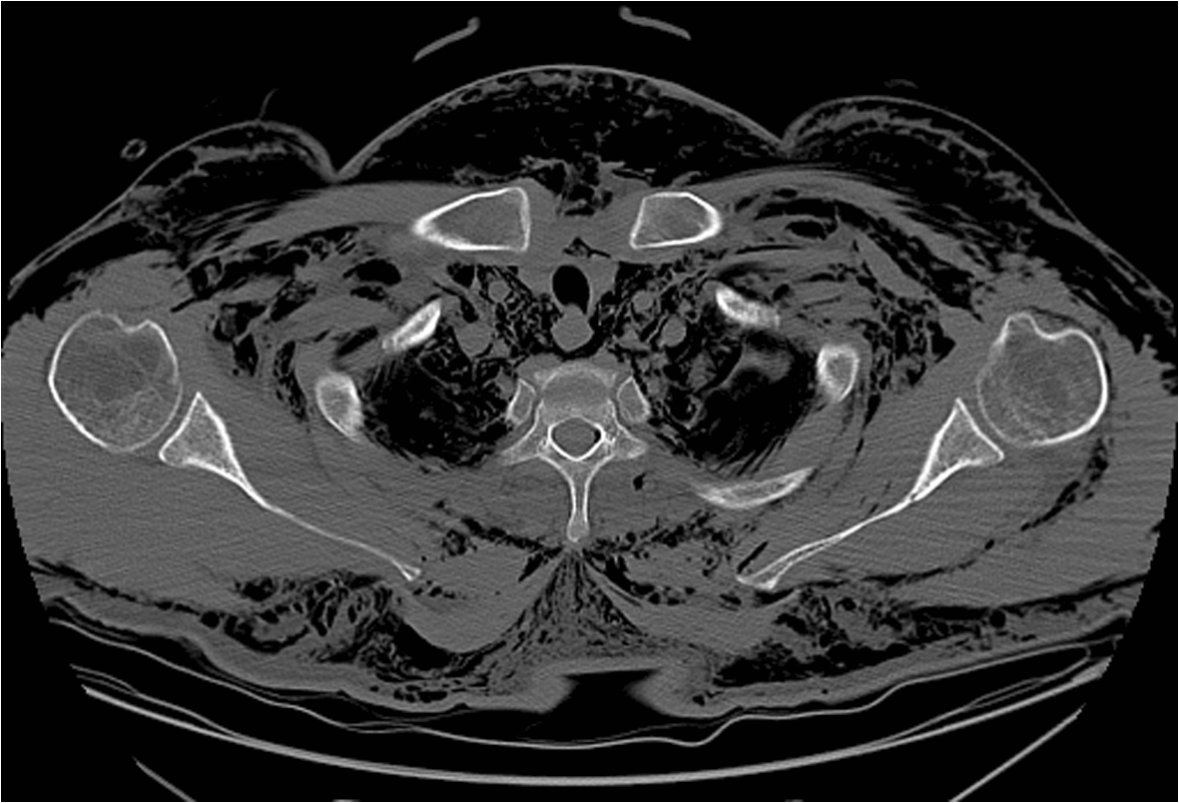
Axial chest computerized tomography scan demonstrating subcutaneous free air throughout the chest secondary to a pneumothorax.

The orthopaedics team was consulted to evaluate his amputated fingers, the foreign body in his toe and to urgently rule out a necrotizing soft tissue infection. His amputations were treated non-operatively and the nail was removed without complication. The subcutaneous emphysema was very concerning for a necrotizing soft tissue infection, especially given his history with traumatic open wounds, though was not a classic presentation for necrotizing fasciitis. His skin wounds had occurred weeks before presentation, his extremity pain was minimal, he did not have thrombotic changes in the skin and his physical exam demonstrated no signs of infection. Further questioning revealed the emphysema had been present since his blunt chest injuries, and though significant, was not consistent with a necrotizing infection. Close review of all imaging studies, specifically plain radiographs and a CT scan, demonstrated subcutaneous emphysema throughout the corpus in a pattern consistent with a causal mechanism of pneumothorax, not an infection along the deep fascial planes (Figures 1, 2, 3 and 4).

The Laboratory Risk Indicator for Necrotizing Fasciitis (LRINEC) score was calculated from laboratory values [8]. The patient received 4 points for a CPR >150 mg/L, one point for a WBC between 15 and 25, one point for a hemoglobin between 11 and 13.5 g/dL, two points for a sodium <135 mmol/L, 2 points for Creatinine of > 141 μmol/L, 0 points for glucose < 10 mmol/L. This equated to a total of 10 points on the LRINEC scoring system, highly concerning for a necrotizing infection.

**Figure 5:**
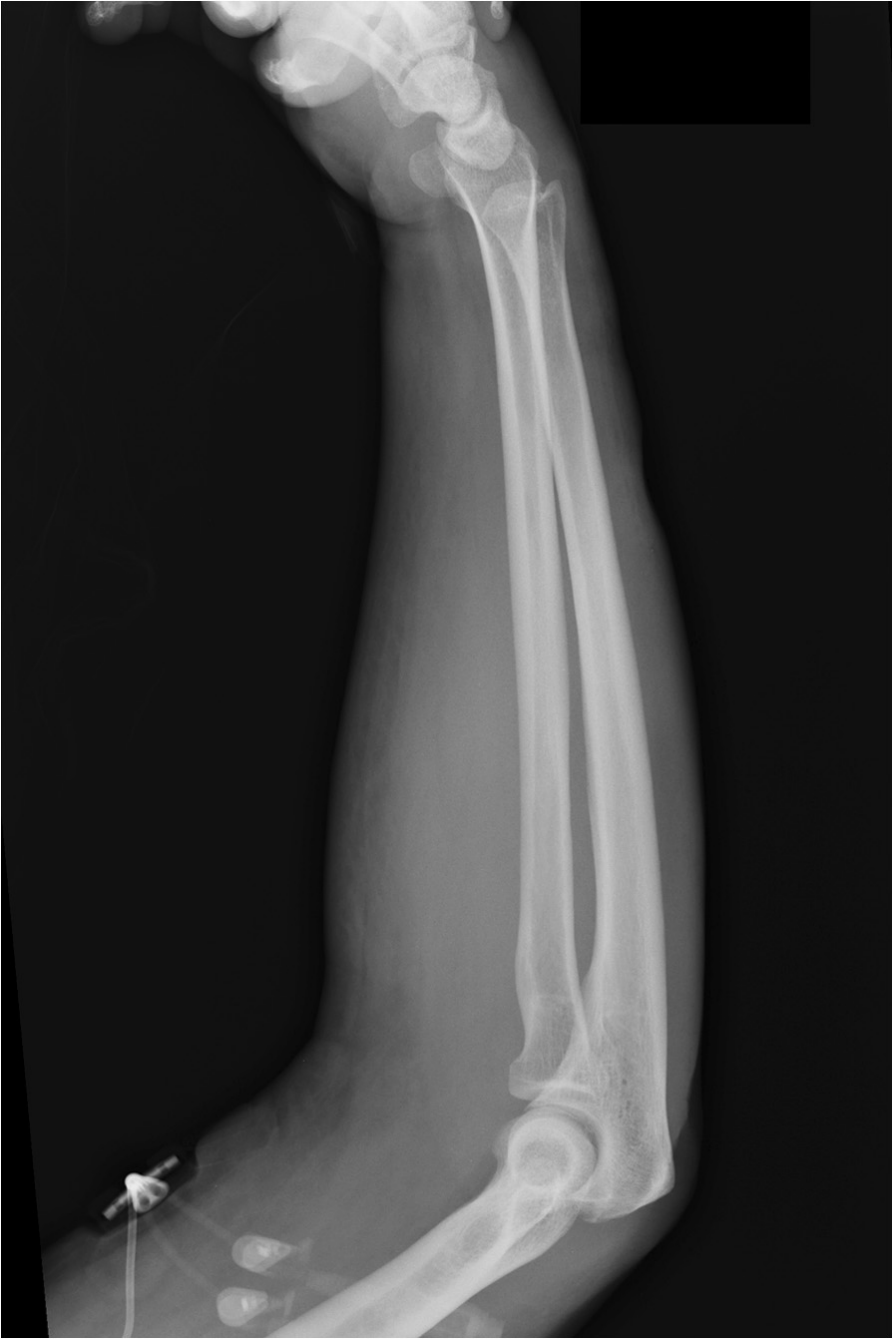
Lateral view of the right arm taken eight days after initial presentation demonstrating resolution of subcutaneous free air.

After thorough evaluation of the patient, imaging, and diagnostics, the subcutaneous emphysema was treated as a non-infectious process. A diagnosis of pneumothorax with associated subcutaneous emphysema secondary to traumatic rib fractures rather than necrotizing fasciitis was made, and the patient was treated with a chest tube and close observation. He subsequently underwent surgical fixation of his mandible fracture and continued non-operative treatment of his upper and lower extremity injuries. The subcutaneous emphysema resolved by hospital day eight with normalization of his clinical and radiographic exams and his wounds remained clean without signs of infection. (Figure 5) While aggressive surgical and non-surgical management of his multi-system traumatic injuries were appropriately performed, worsening of both renal and liver function complicated the clinical course for this patient. On hospital day 11 he died, and though no post-mortem autopsy was performed, death was attributed to multisystem organ failure secondary to his delayed poly-trauma presentation and sepsis.

Subcutaneous emphysema has many causes ranging from the benign and non-infectious to severe necrotizing infections. In evaluating patients with subcutaneous emphysema a wide differential diagnosis is required to appropriately identify and treat the cause. Non-infectious causes of subcutaneous emphysema include trauma, perforation of the pulmonary or digestive system, blast injuries, air gun injuries, high pressure injection injuries, factitious injection of air, cutaneous ulcers, elbow arthroscopy, dental extraction, and iatrogenic use of hydrogen peroxide [1-5]. One case report highlights an episode of benign subcutaneous emphysema of the upper extremity without fractures or a pneumothorax, while another reports on self-induced lower extremity subcutaneous emphysema that was misdiagnosed as necrotizing fasciitis, leading to unnecessary surgical debridement [9,10]. Pediatric surgery literature reports a case of massive subcutaneous emphysema likely due to complication of central line placement and plastic surgery literature reports on noninfectious upper extremity subcutaneous emphysema due to a traumatic wound [6,11]. One defining characteristic of benign emphysema is that it remains confined to the superficial soft tissues while respecting fat pads and tissue planes. Necrotizing fasciitis does not necessarily respect tissue planes and intramuscular air is often present [6,7].

There are other causes of infectious subcutaneous emphysema in an extremity, and providers should consider these when building a differential diagnosis. Both general surgery and orthopaedic literature have reported on bowel perforations as a cause for subcutaneous emphysema of the thigh, hip and throughout the lower extremity [12-25]. These illustrate the importance of determining the cause for the subcutaneous emphysema, whether infectious or not, and involving all appropriate medical and surgical specialists.

Necrotizing fasciitis is one of a handful of true orthopaedic emergencies. This rare infection progresses rapidly and is associated with mortality rates ranging from 6-76% [8,26,27]. Infection spreads rapidly, up to 1 inch per hour, and the best clinical outcomes result from early diagnosis and aggressive surgical debridement of all involved fascia [28,29]. Common etiologies include gas-forming bacteria including Group A beta-hemolytic Streptococcus or Clostridium species, though recent reports include methicillin-resistant Staphylococcus aureus as a causal organism [30]. Diagnosis can be challenging, and the physical examination and laboratory findings can be rather benign with subcutaneous emphysema as the only radiographic findings. Diagnostic tests have been evaluated and the LRINEC adds to the body of evidence when making decisions. Wong et al. found the probability of having a necrotizing infection with a LRINEC score of less than or equal to 5 was a low, moderate with a score of 6–7 and high with a score of greater than or equal to 8. Despite this, clinical judgment must always be applied and other non-infectious causes must also be considered. Despite the importance of early surgical debridement for necrotizing fasciitis and the association of necrotizing fasciitis with subcutaneous emphysema, the presence of subcutaneous emphysema does not preclude the need for proper workup and identification of the source.

## Conclusions

This case illustrates the importance of rapid and appropriate diagnosis of subcutaneous emphysema causal factors. In this case, late diagnosis of a pneumothorax resulted in delayed definitive treatment, which may have contributed to the patient’s ultimate demise. The patient’s presentation following blunt trauma with rib fractures and extensive subcutaneous emphysema was initially misdiagnosed as a necrotizing infection rather than a pneumothorax. This patient sustained significant blunt trauma prior to presentation, and as in all acute and sub-acute trauma, a high level of suspicion for life-threatening injuries must be maintained. Advanced Trauma Life Support (ATLS) teaches an evaluation of patients with the “ABCDE” pneumonic during the primary survey. Utilizing this systematic evaluation the second step would have identified “breathing and ventilation” as the causal factor, allowing appropriate treatment of the underlying pneumothorax. In trauma situations, decision-making for initial treatment should be based on the basic tenants ATLS to primarily address injuries and help prevent further disability or death.

## Consent

The patient died during his hospitalization and therefore cannot be notified or consent to publication of this case report.

## Abbreviations

WBC: White blood cell; CRP: C reactive protein; CT: Computerized tomography; LRINEC: Laboratory risk indicator for necrotizing fasciitis.

## Competing interests

The authors declare they have no competing interests with regard to this publication.

## Authors’ contributions

LS: Primary author, designed and wrote manuscript. PS: Critically edited and assisted in manuscript authorship. KR: Primary surgeon and patient care giver. Collected data and edited manuscript. AS: Assisted in patient care, data collection and critically editing manuscript. All authors read and approved the final manuscript.
